# Acute otitis media complicated by mastoiditis and otogenic transverse-sigmoid sinus thrombosis in an adult: a case report on infectious pathogenesis and multidisciplinary therapy

**DOI:** 10.3389/fmed.2026.1873198

**Published:** 2026-08-24

**Authors:** Fabián Darío Arias Rodríguez, Mercedes Larenas, María Roxana Gastañaga, María Eva Casal, Mónica Maya-Castro, Juan S. Izquierdo-Condoy

**Affiliations:** 1 Departamento de Otorrinolaringología, Hospital Dr. Ricardo Gutiérrez de La Plata, La Plata, Argentina; 2 Department of Otolaryngology, Faculty of Medicine, Universidad de las Americas, Quito, Ecuador; 3 One Health Research Group, Universidad de las Americas, Quito, Ecuador

**Keywords:** acute otitis media, anticoagulation, antimicrobial therapy, cerebral venous sinus thrombosis, mastoiditis, otogenic infection, septic thrombophlebitis, surgical source control

## Abstract

**Background:**

Acute otitis media is usually a self-limited bacterial infection; however, in rare cases, contiguous spread to the mastoid and adjacent dural venous sinuses may result in life-threatening intracranial complications. Otogenic transverse-sigmoid sinus thrombosis is uncommon in adults and represents a diagnostic and therapeutic challenge because its early presentation may be nonspecific and evidence guiding management remains limited.

**Case presentation:**

We report the case of a 32-year-old man with no relevant medical history who presented with a 3-week history of right-sided otorrhea and otalgia, with clinical worsening despite outpatient amoxicillin-clavulanic acid. Physical examination revealed purulent otorrhea, external auditory canal edema, a bulging tympanic membrane, postauricular erythema, tenderness, and a positive Jacques sign. Contrast-enhanced computed tomography demonstrated acute mastoiditis with erosion of the sigmoid plate and thrombosis of the right transverse-sigmoid venous complex extending into the ipsilateral internal jugular vein. Magnetic resonance venography confirmed absence of venous filling in the affected venous territory. The patient was managed using a stepwise multidisciplinary approach that included broad-spectrum intravenous antibiotics, anticoagulation with enoxaparin, myringotomy with ventilation tube placement, mastoidectomy, lateral sinus puncture, and drainage of a mastoid tip abscess. Microbiological cultures obtained after antibiotic exposure showed no growth. The patient had a favorable short-term clinical and otologic course, with mild residual conductive hearing loss at 1 month; however, postoperative vascular imaging was not available to objectively document venous recanalization.

**Conclusion:**

This case illustrates the pathophysiological continuum from acute otogenic infection to contiguous mastoid involvement, sigmoid plate erosion, presumed septic venous thrombosis, and potential intracranial extension. It also highlights the importance of early contrast-enhanced imaging, targeted venous assessment, antimicrobial escalation, surgical source control, individualized anticoagulation, and structured follow-up in adults with complicated otogenic infections. Because microbiological confirmation and postoperative vascular imaging were not available, the infectious and thrombotic interpretation should be considered clinically inferred rather than directly demonstrated.

## 1 Introduction

Acute otitis media (AOM) is one of the most common infections of the middle ear and, in most cases, follows a favorable course with timely treatment. However, in a small proportion of patients, the infection may spread to adjacent structures and lead to extracranial and intracranial suppurative complications, including mastoiditis, dural venous sinus thrombosis, and intracranial abscesses (1, 2). Although these complications are currently uncommon, their clinical relevance is unquestionable because of their potential severity and the possibility of neurological and otological sequelae if diagnosis and treatment are delayed. In this context, imaging studies and early management with broad-spectrum antibiotics, with or without surgical intervention, remain fundamental pillars for limiting disease progression and improving prognosis (3).

Otogenic thrombosis of the lateral sinus or the transverse-sigmoid sinus complex is a rare complication reported more frequently in pediatric populations, although it also occurs in adults, in whom it may go unrecognized because of low clinical suspicion and nonspecific initial presentations (2, 4). Its estimated incidence is low in both AOM and acute mastoiditis, although some studies have documented a higher frequency in selected mastoiditis series; moreover, it is associated with potentially severe outcomes, including neurological deficits, visual disturbances, and mortality (5). The pathophysiology is related to the uncommon contiguous spread of the inflammatory process from the middle ear and mastoid to the dural venous sinuses, thereby promoting thrombosis, venous congestion, increased intracranial pressure, and, in advanced cases, cerebral ischemic compromise (6).

Despite its importance, the management of otogenic transverse-sigmoid sinus thrombosis remains challenging, particularly in adults, in whom the available evidence is limited and clinical suspicion may be lower than in pediatric populations. Key areas of uncertainty include the optimal timing of venous imaging, the interpretation of radiological markers of intracranial extension, the role and duration of anticoagulation, and the timing of surgical source control (7). In this context, we present a case of acute otitis media complicated by mastoiditis, sigmoid plate erosion, transverse-sigmoid sinus thrombosis, and internal jugular vein extension in an adult patient. This report aims to highlight the infectious pathogenesis of contiguous otogenic spread and to discuss the diagnostic and therapeutic decisions required for multidisciplinary management.

## 2 Case presentation

A 32-year-old man with no documented relevant past medical history presented with a 3-week history of right-sided otorrhea, associated with otalgia that had intensified during the 48 h prior to admission. He had previously received outpatient treatment with amoxicillin/clavulanic acid 875/125 mg orally every 12 h for 7 days, without clinical improvement, with persistent otalgia and otorrhea. On physical examination at admission, the right mastoid region was erythematous, with mild fluctuance on palpation and a positive Jacques sign (clinical finding characterized by edema, erythema and effacement of the retroauricular sulcus due to inflammation of the mastoid cells) (Figure 1A). Otoendoscopy revealed edema of the external auditory canal, whitish otorrhea, and a bulging tympanic membrane in the right ear (Figure 1B). Due to symptom progression and findings on physical examination, he was hospitalized with a presumptive diagnosis of acute otitis media complicated by mastoiditis. No ear secretion culture was obtained before admission, and the diagnostic yield of subsequent microbiological testing was considered potentially limited because the patient had already received outpatient antibiotic therapy.

**Figure 1:**
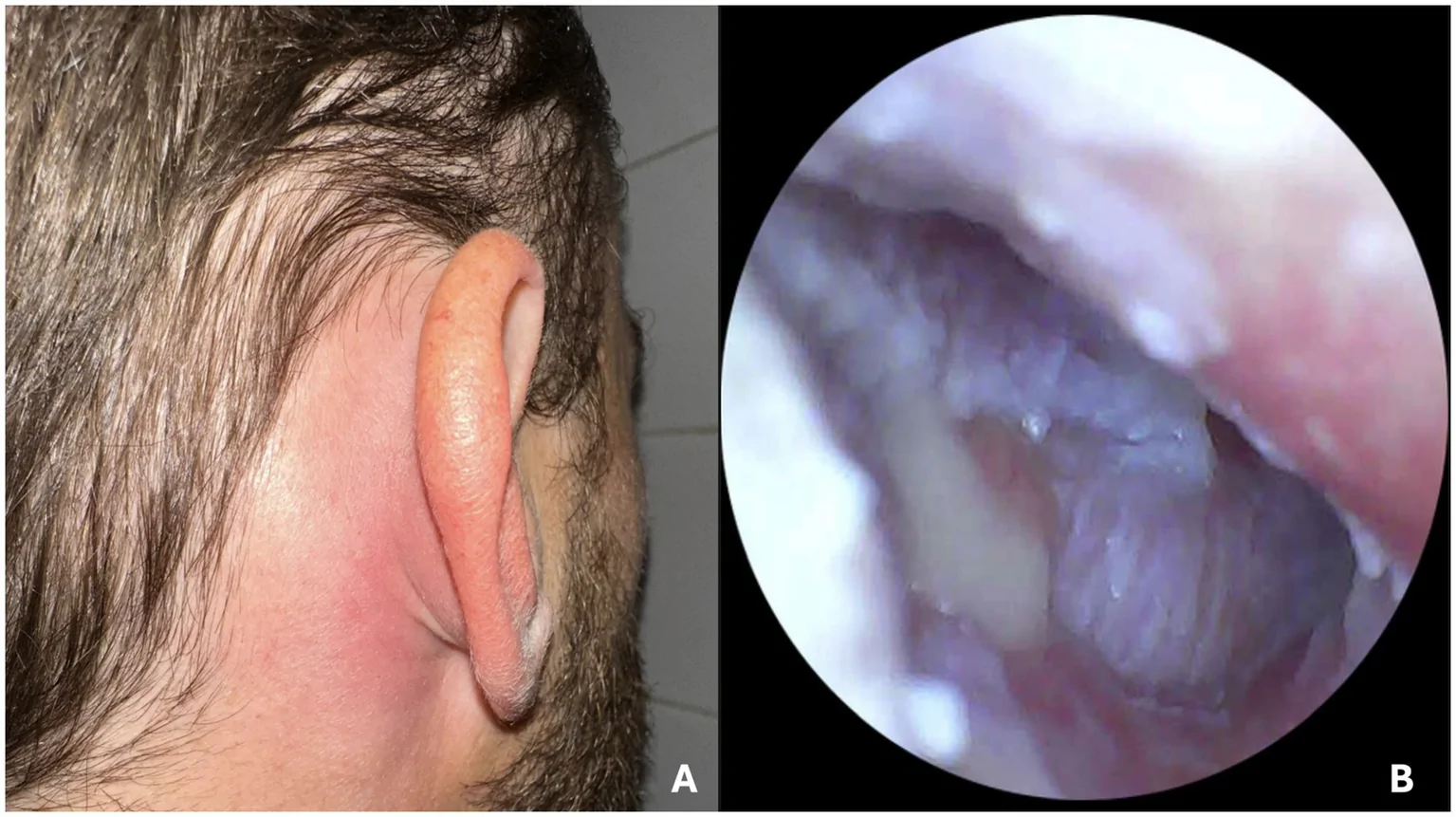
Initial clinical findings in the right ear. **(A)** Right mastoid region with erythema, edema, and a positive Jacques sign, suggestive of acute mastoid involvement. **(B)** Otoendoscopy of the right ear showing edema of the external auditory canal, whitish otorrhea, and a bulging tympanic membrane, findings consistent with complicated acute otitis media.

At admission, the patient was alert and oriented and reported no headache or visual symptoms. No cranial nerve deficits, focal neurological deficits, seizures, or altered mental status were identified. Fundoscopic examination was not performed; therefore, the presence or absence of papilledema could not be objectively assessed. Lumbar puncture was not performed because there were no clinical findings suggestive of meningitis and the diagnosis and extent of venous thrombosis had already been established by contrast-enhanced CT and MRV; therefore, lumbar puncture was not considered necessary for diagnostic or therapeutic decision-making. Initial laboratory tests showed leukocytosis of 20,080 cells/μL with neutrophilia of 18,300 cells/μL and a C-reactive protein level of 120.6 mg/L (Table 1).

**Table 1:** Serial evolution of inflammatory and hematological parameters during hospitalization.

Parameter	Day 1	Day 8	Day 10	Day 14	Day 18	Day 20	Day 24
WBC (×10^3^ cells/μL)	20.08	23.73	30.98	15.57	20.93	23.82	18.72
Neutrophils (×10^3^ cells/μL)	18.30	21.40	27.90	12.84	17.12	18.91	13.29
CRP (mg/L)	120.6	—	—	—	—	—	—

WBC, white blood cells; CRP, C-reactive protein. CRP was only available at baseline; therefore, longitudinal CRP kinetics could not be assessed.

Contrast-enhanced computed tomography of the temporal bone demonstrated opacification of the right middle ear and mastoid air cells by soft-tissue density material, associated with bony erosion of the mastoid septa and the inner mastoid wall (sigmoid plate). Following intravenous contrast administration, a filling defect consistent with thrombosis of the right transverse-sigmoid venous complex was identified, with extension into the ipsilateral internal jugular vein (Figure 2). Dedicated CT venography could not be performed because of institutional equipment limitations.

**Figure 2:**
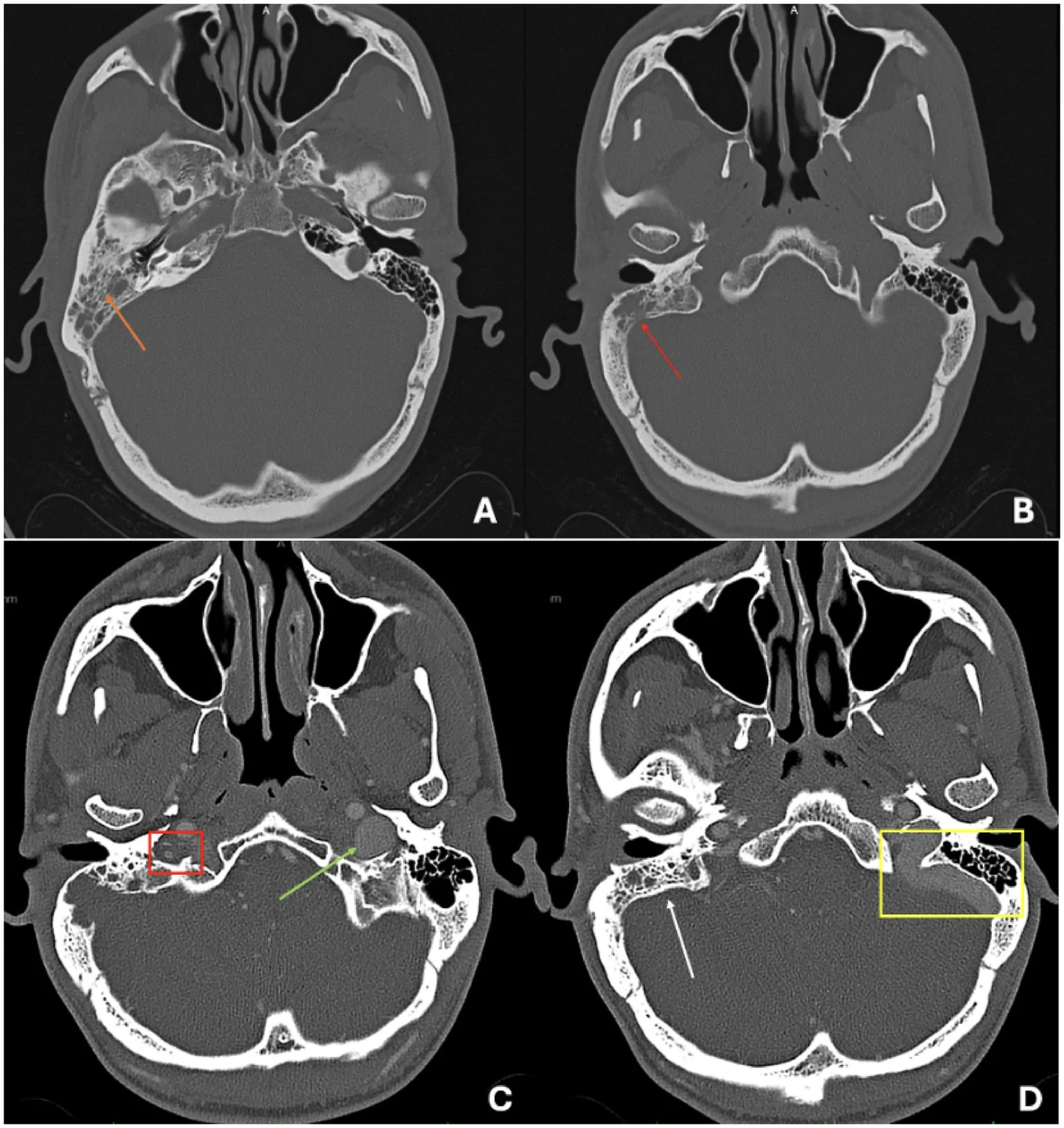
Computed tomography (CT) of the right temporal bone. **(A)** Axial non-contrast CT in bone window showing opacification of the right mastoid air cells by soft-tissue density material (orange arrow). **(B)** Axial non-contrast CT in bone window showing erosion of the right mastoid sigmoid plate (red arrow). **(C)** Axial contrast-enhanced CT showing a filling defect in the right Internal jugular vein (red box). **(D)** Axial contrast-enhanced CT showing a filling defect in the right Transverse-sigmoid venous complex (white arrow), consistent with otogenic venous thrombosis.

During hospitalization, empirical intravenous antimicrobial therapy was initiated with ampicillin/sulbactam 1.5 g every 6 h plus metronidazole 500 mg every 8 h for 5 days, providing broad-spectrum coverage for complicated otogenic infection, including anaerobic organisms. The regimen was subsequently changed to ceftriaxone 2 g intravenously every 12 h, which was administered for 7 days before transfer. Antimicrobial selection was guided by the clinical severity of the infection, available recommendations for complicated otogenic infection, and local antimicrobial availability. After radiological confirmation of thrombosis involving the right transverse-sigmoid venous complex with extension into the ipsilateral internal jugular vein, anticoagulation with enoxaparin 60 mg subcutaneously every 12 h was initiated on hospital day 8 following multidisciplinary assessment. Anticoagulation was continued for 14 days and was discontinued after clinical improvement and intraoperative lateral sinus puncture did not reveal an accessible clot. Nevertheless, this intraoperative finding was interpreted cautiously, since absence of clot on puncture does not exclude residual mural thrombosis, distal thrombus, or incomplete venous recanalization.

On hospital day 9, magnetic resonance venography was performed, confirming an acute inflammatory process in the right mastoid region and demonstrating absence of venous filling in the right transverse sinus and the ipsilateral internal jugular vein, consistent with otogenic venous thrombosis (Figure 3). On day 12, myringotomy with placement of a transtympanic ventilation tube was performed; no middle ear fluid content was obtained during the procedure.

**Figure 3:**
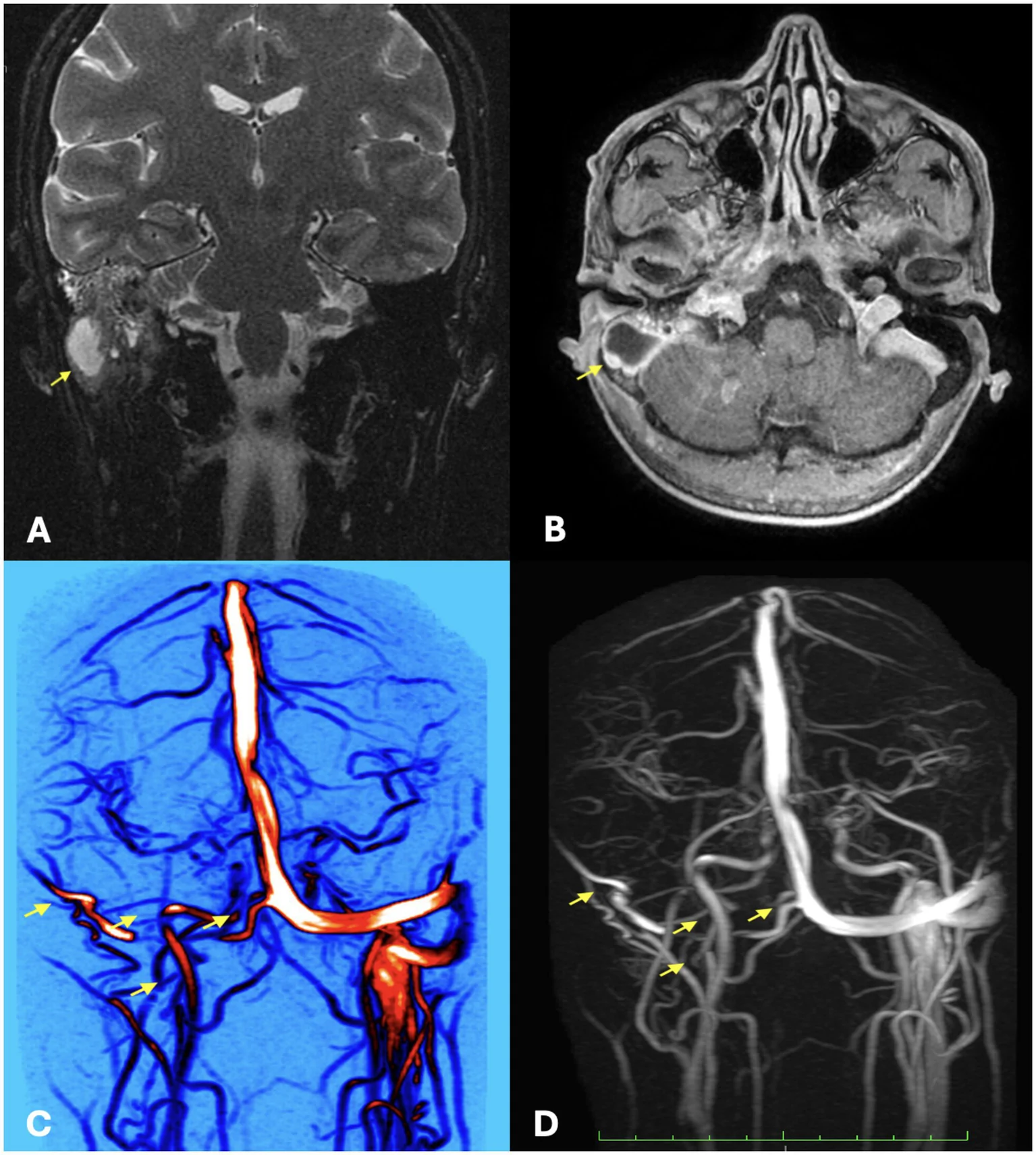
Magnetic resonance imaging and venography of the right mastoid and intracranial venous territory. **(A)** Coronal T2-weighted fast spin-echo MRI showing inflammatory changes and fluid signal in the right mastoid region (yellow arrow). **(B)** Axial post-gadolinium fat-suppressed T1-weighted MRI showing enhancement of the right mastoid and adjacent soft tissues (yellow arrow). **(C)** Three-dimensional time-of-flight magnetic resonance venography (3D TOF-MRV) with volume-rendered reconstruction. **(D)** Three-dimensional TOF-MRV with maximum-intensity-projection reconstruction. **(C,D)** Demonstrate absence of the expected venous signal in the right transverse-sigmoid venous territory and ipsilateral internal jugular vein (yellow arrows), consistent with otogenic venous thrombosis.

On hospital day 13, the patient was transferred to a higher-complexity center with intensive care and neurological monitoring capabilities. Ceftriaxone 2 g intravenously every 12 h was continued at the receiving center to complete a total of 14 days of ceftriaxone therapy and 19 days of intravenous antimicrobial therapy overall. The final antimicrobial dose was administered on hospital day 20. On hospital day 20, a simple right mastoidectomy and drainage of the mastoid tip abscess were performed to achieve definitive surgical source control. Lateral sinus puncture was performed intraoperatively to assess for an accessible intraluminal thrombus that might require direct surgical management; no clot was obtained. Samples obtained during surgery were sent for microbiological, mycological, and pathological examination, with no microbiological growth or specific pathological findings.

Clinical improvement was assessed using clinical, neurological, laboratory, and otologic criteria. Clinically, the patient showed resolution of otalgia, purulent otorrhea, and postauricular inflammatory findings. Neurologically, he remained alert and oriented, without new cranial nerve abnormalities, focal neurological deficits, seizures, or altered mental status. Leukocyte and neutrophil counts decreased from peak values of 30.98 × 10^3^ cells/μL and 27.90 × 10^3^ cells/μL on hospital day 10 to 18.72 × 10^3^ cells/μL and 13.29 × 10^3^ cells/μL on hospital day 24, respectively, although complete hematological normalization was not achieved. Serial CRP kinetics could not be assessed because CRP was available only at baseline.

One month after surgery, pure-tone audiometry demonstrated mild conductive hearing loss in the right ear, with thresholds ranging from 20 to 25 dB. Speech audiometry showed a voice detection threshold of 20 dB, a speech threshold of 25 dB, and 100% maximum discrimination at 30 dB. During subsequent outpatient follow-up, with quarterly evaluations in the otology clinic, the patient showed sustained clinical and endoscopic improvement, with a dry ear, absence of purulent discharge, and a properly positioned transtympanic ventilation tube (Figure 4). No recurrent otorrhea, postauricular inflammatory signs, or clinically evident neurological deterioration were documented during the available follow-up. Postoperative MRV or Doppler ultrasound could not be performed because of institutional and logistical limitations; therefore, venous recanalization could not be objectively assessed (Table 2).

**Figure 4:**
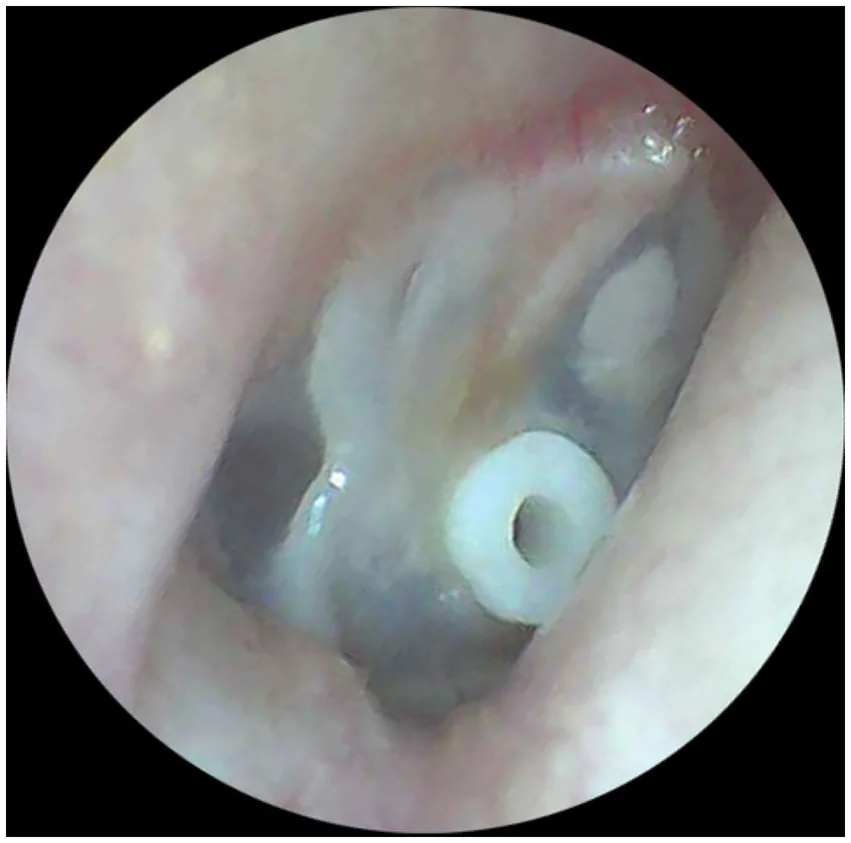
Postoperative otoendoscopic follow-up of the right ear. A dry ear is observed, without purulent discharge, with the presence of cerumen and a properly positioned transtympanic ventilation tube, findings consistent with local clinical improvement during outpatient follow-up.

**Table 2:** Clinical timeline of presentation, diagnostic evaluation, treatment, and follow-up.

Time point	Clinical event
~3 Weeks before admission	Onset of right-sided otorrhea and otalgia.
7 Days before admission	Outpatient amoxicillin/clavulanate 875/125 mg orally every 12 h for 7 days; persistent otalgia and otorrhea without clinical improvement.
Hospital day 1	Hospital admission for suspected acute otitis media complicated by mastoiditis; initial laboratory evaluation and contrast-enhanced temporal bone CT; intravenous ampicillin/sulbactam plus metronidazole initiated.
Hospital days 1–5	Ampicillin/sulbactam 1.5 g every 6 h plus metronidazole 500 mg every 8 h.
Before transfer	Ceftriaxone 2 g intravenously every 12 h was administered for 7 days.
Hospital day 8	Therapeutic anticoagulation with enoxaparin 60 mg subcutaneously every 12 h initiated after confirmation of transverse-sigmoid sinus thrombosis with internal jugular vein extension.
Hospital day 9	MRI/MRV confirmed absence of venous filling in the right transverse-sigmoid venous territory and ipsilateral internal jugular vein.
Hospital day 12	Myringotomy and transtympanic ventilation tube placement; no middle ear fluid obtained.
Hospital day 13	Transfer to a higher-complexity center for intensive care and neurological monitoring; ceftriaxone continued.
After transfer	Ceftriaxone was continued at the receiving center to complete 14 days of ceftriaxone therapy and 19 days of intravenous antimicrobial therapy overall; the final antimicrobial dose was administered on hospital day 20.
Hospital day 20	Simple right mastoidectomy, drainage of mastoid tip abscess, and lateral sinus puncture; no accessible clot obtained. Final day of intravenous antimicrobial treatment.
14-Day anticoagulation period	Enoxaparin administered for a total of 14 days and subsequently discontinued after multidisciplinary reassessment and clinical improvement.
1 month after surgery	Pure-tone audiometry showed mild right-sided conductive hearing loss of 20–25 dB.
Subsequent follow-up	Quarterly otology evaluations showed a dry ear, no recurrent purulent otorrhea, and no clinically evident neurological deterioration. Postoperative vascular imaging could not be obtained.

## 3 Discussion

AOM usually resolves favorably with timely treatment; however, in a small proportion of cases, it may extend to adjacent structures and cause extracranial and intracranial complications, such as mastoiditis, intracranial abscesses, and otogenic venous thrombosis of the transverse-sigmoid complex (1, 2). Although uncommon, these complications remain clinically relevant because of their potential association with neurological and otological sequelae, particularly when diagnosis and treatment are delayed (3, 7). In this case, progression from AOM with persistent otorrhea to mastoiditis and thrombosis of the right transverse-sigmoid venous complex, with extension into the ipsilateral internal jugular vein, illustrates an unusual but clinically significant form of complicated otogenic disease.

The novelty of this case does not rely solely on the rarity of otogenic venous thrombosis, but on the integration of anatomical, radiological, infectious, and therapeutic elements in an adult patient: persistent otogenic infection, sigmoid plate erosion, transverse-sigmoid sinus thrombosis, internal jugular vein extension, empirical antimicrobial escalation, individualized anticoagulation, and definitive surgical source control.

From an epidemiological perspective, lateral sinus thrombosis accounts for only a small proportion of intracranial complications associated with middle ear infections, estimated at less than 6% in some series (4). More recent studies on otogenic cerebral venous thrombosis have reported an approximate incidence of 0.67 per 100,000 cases of AOM and 1.6 per 100,000 cases of acute mastoiditis, although in selected cohorts it may be observed in up to 2.7% of mastoiditis cases; moreover, it has been associated with severe outcomes, including motor deficits, visual disturbances, and mortality rates ranging from 9 to 29% (5, 8, 9). Although this entity has been described predominantly in the pediatric population, its occurrence in adults should not be underestimated, as the lower level of clinical suspicion in this age group may favor diagnostic and therapeutic delays (2, 4, 8). In the present patient, the absence of relevant past medical history and the initial presentation centered on local otological findings reinforce precisely the need to maintain a high index of suspicion in adults with mastoiditis and an unfavorable clinical course.

Adult-specific evidence on otogenic venous sinus thrombosis remains limited. Huang et al. compared 17 adults with 10 pediatric patients with otogenic lateral sinus thrombosis and reported that otorrhea was more frequent and baseline hearing impairment was greater among adults (10). Wierzbicka et al., in a series of adult intracranial otogenic complications, identified sigmoid sinus thrombosis as an important component of complicated disease, with headache and, in some patients, neurological manifestations such as altered consciousness, facial nerve paresis, or seizures (11). In contrast, our patient developed extensive transverse-sigmoid thrombosis with internal jugular vein extension despite the absence of headache, visual symptoms, focal neurological deficits, seizures, or altered mental status. This predominantly otologic presentation illustrates that substantial venous extension may occur in adults without overt neurological manifestations. Similar to the adult case reported by Dao et al., the present case also underscores the need to consider venous thrombosis when acute mastoiditis follows an unfavorable clinical course (3). Collectively, these adult observations emphasize the clinical relevance of maintaining a low threshold for venous imaging in complicated mastoiditis.

The clinical course also raises the possibility of partially treated or “masked” mastoiditis. The patient had persistent otorrhea for approximately 3 weeks and had received outpatient amoxicillin-clavulanic acid before referral, yet the infection progressed to coalescent mastoiditis, sigmoid plate erosion, and transverse-sigmoid sinus thrombosis. Prior or partially effective antibiotic exposure may attenuate overt otologic manifestations without eradicating mastoid inflammation, allowing subclinical progression and delayed recognition of extracranial or intracranial complications. In the present case, the persistent otorrhea and later radiological findings suggest that antimicrobial treatment may have been insufficient in spectrum, dose, duration, source control, or a combination of these factors. Therefore, rather than representing a completely silent disease course, this case is better interpreted as a partially treated otogenic infection with clinically attenuated but progressive mastoid involvement (12).

The pathophysiology of this complication is explained by contiguous spread of the infectious process from the middle ear to the mastoid air cells and subsequently to the sigmoid plate and adjacent dural venous sinuses, thereby promoting thrombosis, venous congestion, and increased intracranial pressure; in advanced cases, impaired venous drainage may even compromise cerebral perfusion and lead to ischemia or infarction (6, 13). This pathophysiological sequence is summarized in Figure 5.

**Figure 5:**
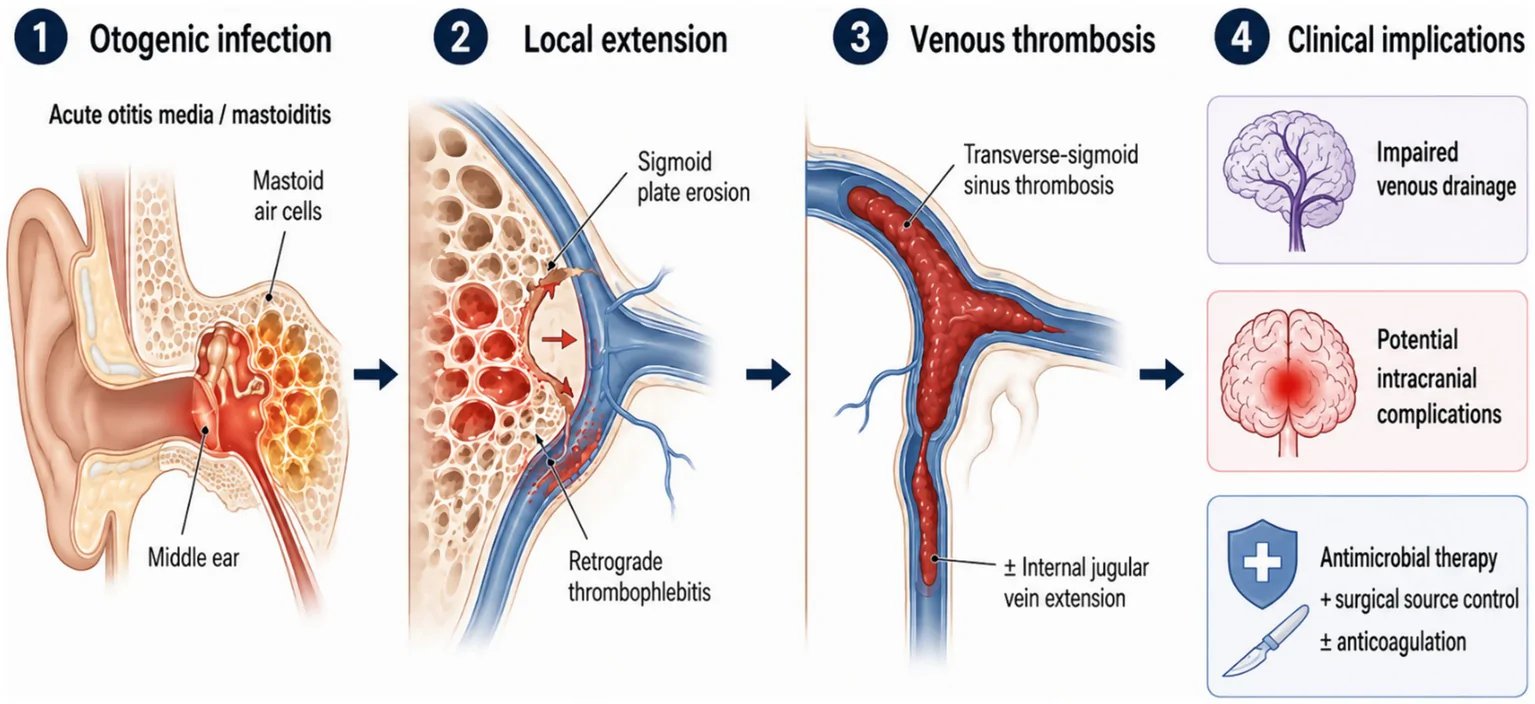
Proposed pathway of otogenic transverse-sigmoid sinus thrombosis. Otogenic infection may progress from acute otitis media to mastoiditis and extend to the adjacent dural venous system through sigmoid plate erosion or retrograde thrombophlebitis. This process may result in transverse-sigmoid sinus thrombosis, with possible extension into the internal jugular vein, leading to impaired venous drainage and potential intracranial complications. Management requires antimicrobial therapy and surgical source control, with individualized consideration of anticoagulation.

From an infectious diseases perspective, this case illustrates a clinically relevant continuum between localized mucosal infection, mastoid air-cell involvement, bony barrier disruption, endothelial injury, and presumed septic venous thrombosis. The erosion of the sigmoid plate observed on contrast-enhanced CT supports direct anatomical progression from the mastoid focus toward the adjacent venous sinus. In this setting, thrombosis should not be interpreted solely as an isolated vascular event, but rather as the probable result of infection-associated inflammation, impaired venous drainage, and local prothrombotic activation. However, because microbiological confirmation of the thrombus was not available, the term septic thrombophlebitis should be understood as a clinically inferred mechanism supported by the anatomical and radiological context, rather than as a directly proven microbiological diagnosis.

This mechanism is consistent with the findings observed in our case, in which contrast-enhanced computed tomography showed mastoid opacification, erosion of the sigmoid plate, and venous filling defects, while magnetic resonance venography confirmed absence of filling in the affected venous territory. Given the CT evidence of mastoid coalescence, sigmoid plate erosion, and venous filling defects, magnetic resonance venography was performed to better characterize the extent of otogenic venous involvement and to exclude additional intracranial complications. This decision was clinically relevant because otogenic venous thrombosis may occur without early neurological manifestations, particularly in adults, and delayed recognition may increase the risk of intracranial hypertension, venous infarction, or persistent septic complications (20).

A further imaging limitation was the unavailability of dedicated CT venography because of institutional equipment constraints. Consequently, venous assessment relied on contrast-enhanced CT and subsequent MR venography.

In this regard, the case highlights the value of contrast-enhanced imaging studies and targeted venous assessment when signs of complicated mastoiditis are present, as the initial clinical picture may be nonspecific and not always accompanied by florid neurological manifestations. Symptoms such as retroauricular pain, edema, mastoid erythema, persistent fever, or clinical worsening despite antibiotic therapy should prompt a more thorough investigation to rule out intracranial extension (2, 3, 8, 13).

A limitation of this case is that fundoscopic examination was not performed to assess papilledema or other signs of intracranial hypertension. Although the patient did not present florid neurological manifestations and venous involvement was characterized by CT and MRV, systematic ophthalmological evaluation may be useful in similar cases, particularly when headache, visual symptoms, persistent vomiting, altered mental status, or radiological findings suggest impaired venous drainage (14). Host susceptibility should also be considered when severe otogenic complications occur in an otherwise young adult. Diabetes mellitus, immunosuppression, metabolic disorders, chronic ear disease, and prothrombotic conditions may contribute to atypical presentation, delayed diagnosis, persistent infection, or thrombotic extension. In the present case, the patient had no documented relevant medical history or obvious predisposing condition at admission. Nevertheless, a systematic evaluation for occult host susceptibility factors or thrombophilia was not performed beyond routine clinical assessment. This represents an additional limitation of the case, because undetected metabolic, immune, or prothrombotic conditions could have contributed to the severity of the presentation. In similar cases, targeted assessment of glycemic status, immune compromise, and thrombophilic risk may be considered, particularly when thrombosis is extensive, recurrent, disproportionate to the local infection, or occurs in the absence of clear anatomical explanation.

In this case, failure of outpatient amoxicillin-clavulanate to control the infection justified escalation to intravenous antimicrobial therapy after hospitalization. Ampicillin/sulbactam plus metronidazole was initially selected empirically to provide broad coverage against common otogenic pathogens, including anaerobic organisms potentially involved in complicated mastoid infection. The subsequent switch to ceftriaxone was made after multidisciplinary reassessment in view of the extensive radiological disease, including coalescent mastoiditis, sigmoid plate erosion, and intracranial venous involvement. Regimen selection and treatment duration were individualized according to disease severity, available recommendations for complicated otogenic infection, local antimicrobial availability, timing of surgical source control, and clinical response (3, 9, 11, 15).

Anticoagulation was added after confirmation of transverse-sigmoid sinus thrombosis with extension into the internal jugular vein. The role of anticoagulation in otogenic venous sinus thrombosis remains controversial because available evidence is derived mainly from case series, retrospective studies, and systematic reviews rather than randomized trials (20). Mather et al. noted that evidence from cerebral venous thrombosis more broadly supports anticoagulation and suggests a potential reduction in adverse outcomes (16). Contemporary guidance for cerebral venous thrombosis generally supports anticoagulation, often for several months depending on provoking factors, bleeding risk, clinical course, and recanalization status (17). However, otogenic septic thrombosis differs from noninfectious cerebral venous thrombosis because eradication of the infectious focus and surgical source control are central components of management. In the present case, enoxaparin was initiated because of extensive transverse-sigmoid thrombosis with internal jugular vein extension. The 14-day duration was individualized after multidisciplinary assessment, clinical improvement, and intraoperative evaluation. Importantly, the absence of clot on lateral sinus puncture should not be interpreted as definitive evidence of complete thrombus resolution, because residual mural thrombus, distal thrombosis, or incomplete recanalization may persist. The lack of postoperative MRV or Doppler ultrasound is therefore a limitation and prevents objective confirmation of vascular recanalization. This case should consequently be interpreted as having a favorable short-term clinical course rather than proven radiological resolution of the thrombotic event (11, 18).

Surgical intervention was also central to management. Myringotomy with ventilation tube placement was initially performed to improve middle ear drainage; however, definitive source control required mastoidectomy and drainage of the mastoid tip abscess. This stepwise approach reflects a key therapeutic principle in complicated infectious diseases: antimicrobial therapy alone may be insufficient when an anatomical septic focus persists. Therefore, the favorable clinical course in this patient was likely related to the integration of antimicrobial escalation, targeted venous assessment, individualized anticoagulation, and surgical source control.

The absence of microbiological growth in intraoperative samples, particularly after prior antibiotic exposure, does not exclude a bacterial etiology. However, it limits the etiological certainty of the case and weakens direct microbiological support for the diagnosis of septic thrombophlebitis. No complementary molecular tests, such as 16S rRNA gene sequencing, broad-range bacterial PCR, or fungal molecular assays, were performed; and histopathological examination did not identify specific bacterial or fungal structures. This should be acknowledged as a methodological limitation. In future similar cases, surgical specimens should ideally be reserved not only for conventional microbiology and histopathology, but also for molecular testing, particularly when cultures are expected to be negative because of previous antibiotic exposure. Such an approach could improve pathogen identification, antimicrobial stewardship, and the academic rigor of culture-negative complicated otogenic infections (9, 11, 17, 19).

Regarding the clinical course, the patient showed favorable short-term clinical and otologic recovery, although mild conductive hearing loss persisted in the affected ear 1 month after the procedure. This finding is important because it reminds us that, even in the absence of major neurological sequelae, intracranial otogenic complications may leave functional otological sequelae that should be documented and followed systematically (9). Therefore, rather than merely confirming the rarity of this entity, this case underscores three clinically useful messages: first, mastoiditis in adults with a torpid course should raise suspicion of intracranial venous extension; second, contrast-enhanced computed tomography and advanced venous imaging are key tools for confirming the anatomical extent of disease; and third, timely, stepwise, and multidisciplinary treatment may help limit progression and improve clinical outcomes (3, 8, 9, 11). Because postoperative vascular imaging was not available, the report cannot establish whether the transverse-sigmoid sinus or internal jugular vein thrombosis recanalized, partially improved, or persisted asymptomatically.

Taken together, this report contributes to the literature on otogenic venous thrombosis in adults, a population less represented than the pediatric population, and highlights the need to continue generating evidence on the optimal timing of venous imaging, the role of anticoagulation, and the timing of surgical treatment in this type of complication (8, 9, 11, 18).

## 4 Conclusion

Otogenic transverse-sigmoid sinus thrombosis should be considered in adults with mastoiditis or persistent otorrhea that worsens despite antimicrobial therapy, even in the absence of overt neurological deficits. Contrast-enhanced CT complemented by venous imaging can define intracranial extension, while management requires coordinated antimicrobial therapy, individualized consideration of anticoagulation, and timely surgical source control. In this case, short-term clinical recovery was favorable, although mild conductive hearing loss persisted and vascular recanalization could not be objectively confirmed.

## Data Availability

The original contributions presented in the study are included in the article/supplementary material, further inquiries can be directed to the corresponding author.
